# Topological Dynamics of Micelles Formed by Geometrically Varied Surfactants

**DOI:** 10.1021/acs.langmuir.2c00230

**Published:** 2022-08-01

**Authors:** Adrian Sanchez-Fernandez, Johan Larsson, Anna E. Leung, Peter Holmqvist, Orsolya Czakkel, Tommy Nylander, Stefan Ulvenlund, Marie Wahlgren

**Affiliations:** † Food Technology, Engineering and Nutrition, Lund University, Box 124, 221 00 Lund, Sweden; ‡ Biofilms Research Center for Biointerfaces and Department of Biomedical Science, Faculty of Health and Society, Malmö University, Per Albin Hanssons Väg 35, 21432 Malmö, Sweden; § European Spallation Source ERIC, P.O. Box 176, 221 00 Lund, Sweden; ∥ Physical Chemistry, Department of Chemistry, Lund University, Box 124, 221 00 Lund, Sweden; ⊥ Institute Laue-Langevin, 71 Avenue des Martyrs, 38000 Grenoble, France; # EnzaBiotech AB, Scheelevägen 22, 22363 Lund, Sweden

## Abstract

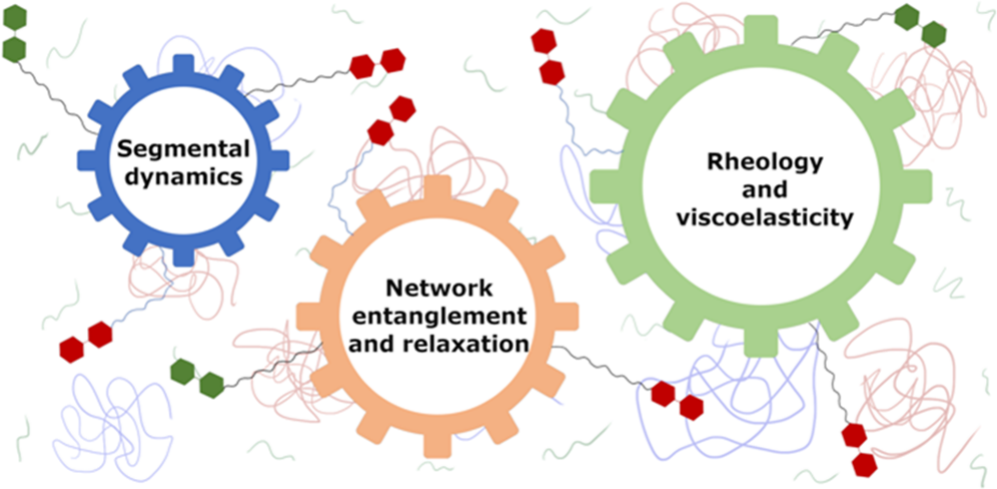

The molecular architecture of sugar-based surfactants strongly affects their self-assembled structure, i.e., the type of micelles they form, which in turn controls both the dynamics and rheological properties of the system. Here, we report the segmental and mesoscopic structure and dynamics of a series of C16 maltosides with differences in the anomeric configuration and degree of tail unsaturation. Neutron spin-echo measurements showed that the segmental dynamics can be modeled as a one-dimensional array of segments where the dynamics increase with inefficient monomer packing. The network dynamics as characterized by dynamic light scattering show different relaxation modes that can be associated with the micelle structure. Hindered dynamics are observed for arrested networks of worm-like micelles, connected to their shear-thinning rheology, while nonentangled diffusing rods relate to Newtonian rheological behavior. While the design of novel surfactants with controlled properties poses a challenge for synthetic chemistry, we demonstrate how simple variations in the monomer structure can significantly influence the behavior of surfactants.

## Introduction

The formation of entangled colloidal networks is of significance in technological processes and formulated products, e.g., for the oil field industry, and for the use in topical drugs and cosmetics.^1^ Worm-like micelles (WLM), often regarded as “living” polymers in the colloid science community, are formed through the self-assembly of surfactants into supramolecular assemblies with a low but positive spontaneous curvature.^2^ Above the overlap concentration, which defines the upper limit of the dilute regime, WLM entangle in solution and the rheological behavior becomes viscoelastic, indicative of gel formation.^3−6^ Although a variety of surfactant mixtures, surfactants in electrolyte solutions, and surfactant–hydrotrope combinations are known to form this type of structure, there is significant ongoing effort to develop sustainable amphiphiles that act as rheological modifiers.^1,2,7−10^ In this context, sugar-based surfactants have emerged as a promising group of amphiphiles.^6−8,11−14^ They have several advantages compared to the other surfactants, mainly: (1) they assemble into WLM without requiring the use of other formulation components, (2) the nonionic character greatly reduces their toxicity and environmental impact, (3) they show a great capacity to withstand temperature and salinity changes, and (4) they can be synthesized using renewable materials.

For hexadecylmaltosides, we have previously shown that changes in the monomer configuration dramatically change their behavior without altering the chemical composition of the surfactant.^12,15^ In particular, the anomeric configuration of the sugar plays a big role in the morphology of the micelle and the rheology of the system (Table 1). While hexadecyl-α-d-maltoside (α-C_16_G_2_) forms short cylindrical micelles, the hexadecyl-β-d-maltoside (β-C_16_G_2_) assembles into long, semiflexible WLM. In the semidilute regime, the entanglement of these β-C_16_G_2_ micelles features viscoelastic, non-Newtonian rheological properties, but the α-C_16_G_2_ system remains Newtonian and shows lower viscosity.^15,16^ Also, when comparing the micelles of β-C_16_G_2_ to those of (*Z*)-hexadec-9-en-1-yl-β-d-maltoside (β-C_16-1_G_2_), a thermally resilient unsaturated analogue, both show almost identical WLM structures.^12^ However, the rheological properties and tensile strength of the unsaturated surfactant solutions are different. As both surfactants form micelles with a very similar structure, we aim to demonstrate that the dynamic behavior of the micelles plays an important role in the response of the system on the macroscopic scale as shown by the rheological behavior.

**Table 1:**
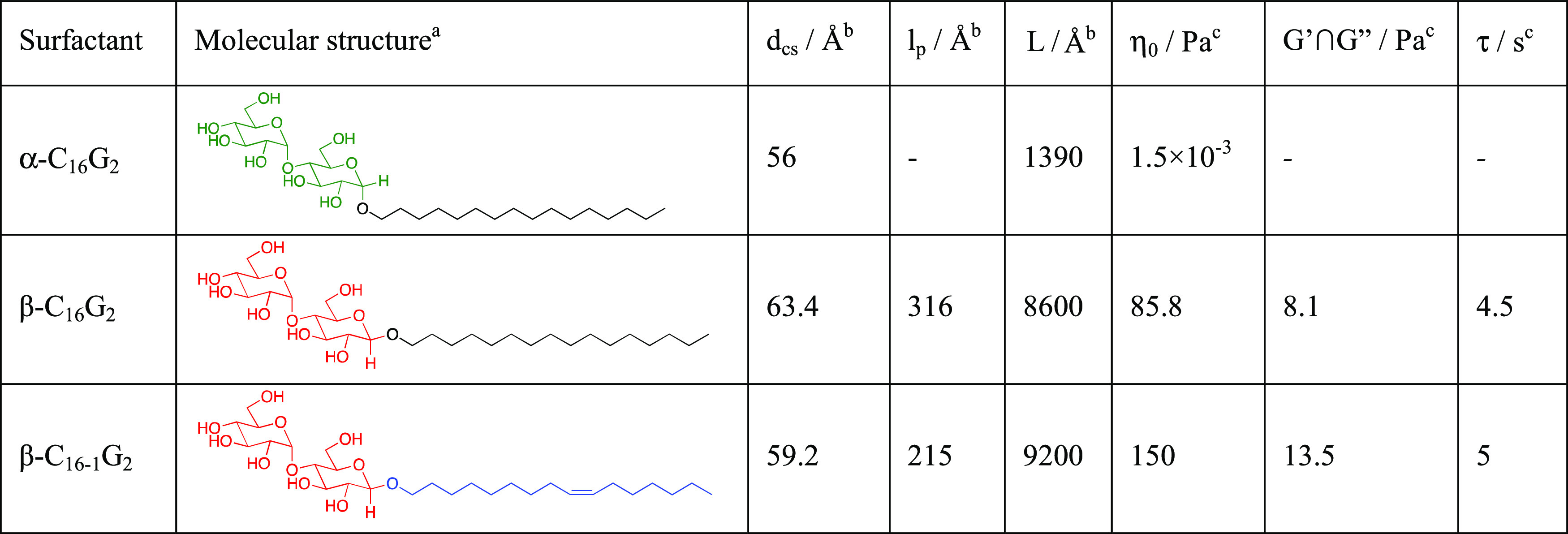
Characteristic Parameters of Micellar Systems from Geometrically Varied Sugar-Based Surfactants^12,15^

a Differences between the monomer structure are highlighted using a color code.

b The structural parameters were derived from the analysis of small-angle scattering data of 10 mM surfactant: *d*_cs_—diameter of the micelle cross section, *l*_p_—persistence length, and *L*—contour length.

c The rheological parameters were determined for 100 mM surfactant concentration: η_0_—zero-shear viscosity; *G*′ ∩ *G*″—intersection between the viscous and elastic modulus, and τ—relaxation time.

A rational approach to the synthesis of surfactants with predictable function requires the relation between monomer chemical structure and its self-assembly to be understood. WLM have a hierarchical structure that can be defined using two characteristic length scales: the segmental length scale of the assembly, which refers to the local structure of micellar segments, and the network length scale, which accounts for the global structure across the contour length of the micelle. As the segmental and network relaxation correlate to the structure and rheology of WLM, dynamic measurements can be used to probe their topological features.^3,17^ Here, neutron spin-echo (NSE) and dynamic light scattering (DLS) are combined to study the topological dynamics of micelles formed by the self-assembly of these three different sugar-based surfactants, namely, α-C_16_G_2_, β-C_16_G_2_, and β-C_16-1_G_2_.

## Results and Discussion

**Figure 1:**
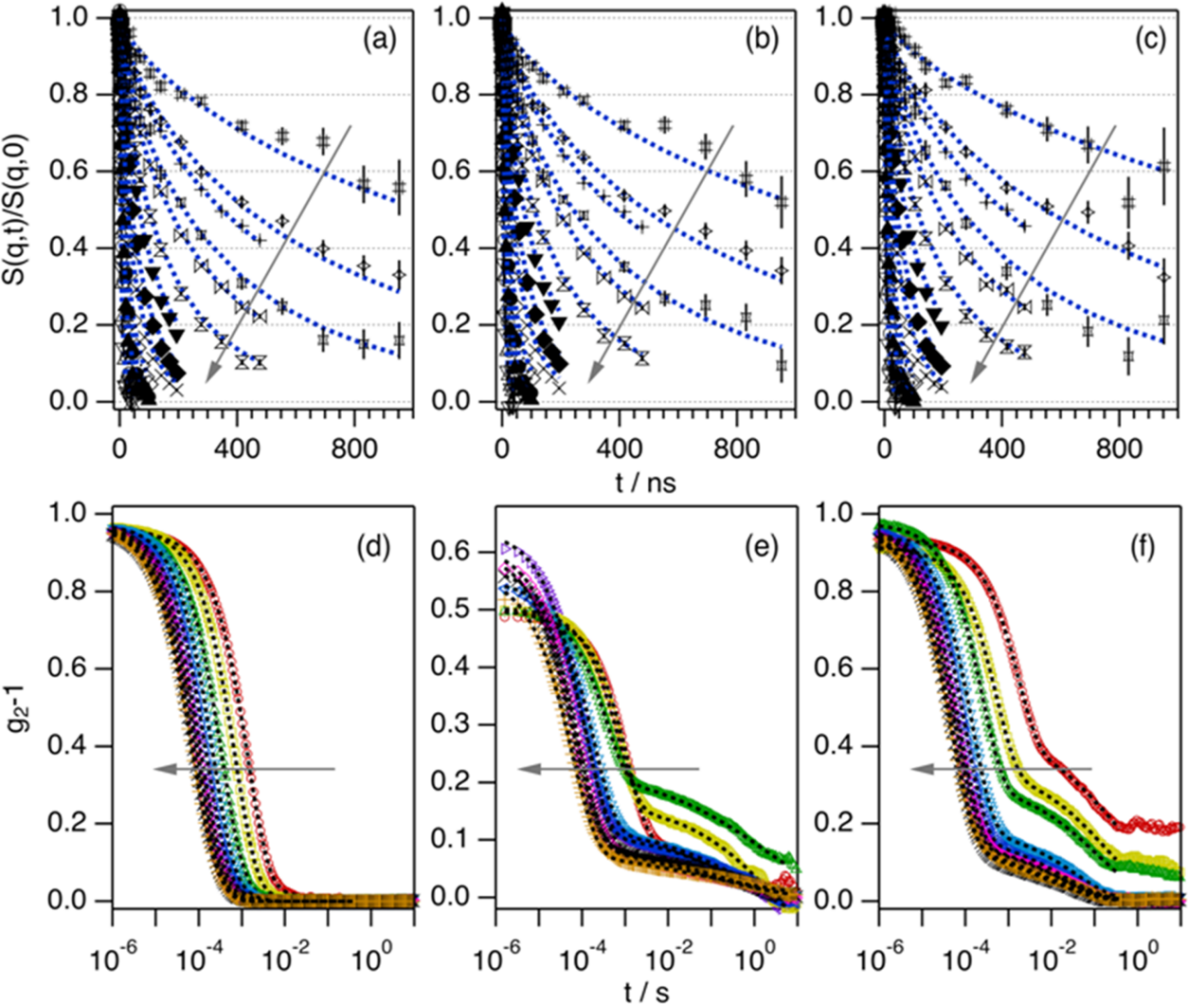
NSE: normalized intermediate scattering functions and best fits for 100 mM of (a) α-C_16_G_2_, (b) β-C_16_G_2_, and (c) β-C_16-1_G_2_ covering a *q*-range between 0.014 and 0.166 Å^–1^. DLS: intensity autocorrelation function for 100 mM (d) α-C_16_G_2_, (e) β-C_16_G_2_, and (f) β-C_16-1_G_2_ covering a *q*-range between 5.48 × 10^–4^ and 2.38 × 10^–3^ Å^–1^. NSE and DLS experiments were performed at 50 °C. Models are presented as dotted lines. The *q*-values increase in the direction of the arrows and are listed in Tables S1 and S2. Where not visible, error bars are within the markers.

The normalized intermediate scattering functions (*S*(*q*, *t*)/*S*(*q*, 0)) were collected on the NSE instrument IN15 at ILL. The obtained *S*(*q*, *t*)/*S*(*q*, 0) were analyzed using a single stretched exponential function, as proposed by Zilman and Granek^18,19^
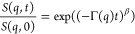
1where Γ(*q*) is the relaxation rate and β is the stretched exponent. Data for β-C_16_G_2_ were initially analyzed using three different approaches: (1) the β value is fitted to the data; (2) fixing β to 3/4, which describes the dynamic behavior of one-dimensional semiflexible chains; and fixing β to 2/3, which is associated with the relaxation of two-dimensional flexible membranes.^3^ The fitted value of the stretched exponent to the data gives an average β of 0.82 ± 0.09 for α-C_16_G_2_, 0.81 ± 0.07 for β-C_16_G_2_, and 0.86 ± 0.07 for β-C_16-1_G_2_. For the values predicted in the theoretical framework, the goodness-of-fit, parametrized in the χ^2^ map, suggests that a stretched exponent of 3/4 is more appropriate than β = 2/3 to describe the relaxation dynamics in the segmental and subsegmental length scales (see Figure S2). These observations agree with previous structural investigations that showed the formation of one-dimensional (1D) micelles for these surfactants.^12,15^ However, the consistently higher values of β obtained may suggest that some relaxation mechanisms associated to 1D semiflexible objects are suppressed, for instance, through topological restraints due to excluded volume effects. The main differences between the fitted β values for the three surfactants are observed at low *q* (0.70 ± 0.05 for α-C_16_G_2_, 0.72 ± 0.07 for β-C_16_G_2_, and 0.81 ± 0.04 for β-C_16-1_G_2_ between 0.0143 and 0.0268 Å^–1^), while at high *q*, these are relatively similar (0.88 ± 0.04 for α-C_16_G_2_, 0.88 ± 0.06 for β-C_16_G_2_, and 0.86 ± 0.05 for β-C_16-1_G_2_ between 0.0836 and 0.166 Å^–1^). The higher β values for the unsaturated surfactant suggest a different relaxation mechanism at this length scale compared to the saturated surfactants. For subsequent analysis of the NSE data, we decided to use the average of the fitted stretched exponents in the Zilman and Granek model. Data and the resulting fits are presented in Figures 1a–c and S1.

**Figure 2:**
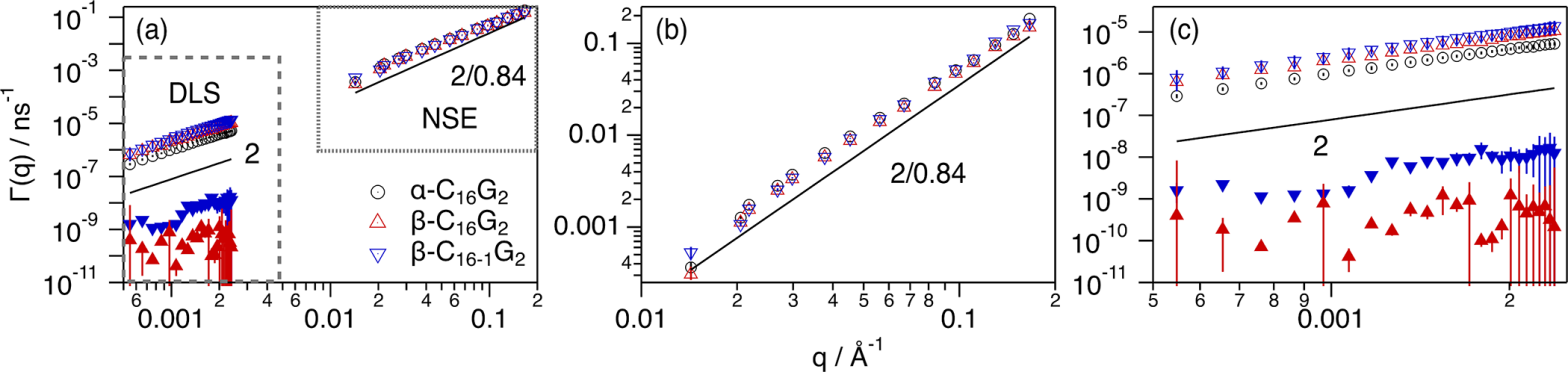
(a) Γ(*q*) vs *q* for the micelle dynamics for the different surfactants as shown in the legend of the graph. (b) Relaxation rates obtained from the NSE analysis covering a *q*-range between 0.014 and 0.166 Å^–1^. (c) Fast (open markers) and slow (filled markers) relaxation rates of the network obtained from the DLS analysis covering a *q*-range between 5.48 × 10^–4^ and 2.38 × 10^–2^ Å^–1^. The solid line shows the scaling expected for the relaxation rates for (b) one-dimensional segmental diffusion, ∝*q*^8/3^, and (c) diffusive mode, ∝*q*^2^. Where not seen, error bars are within the markers.

The dynamics probed using NSE cover the contributions of the cross-sectional fluctuations of the micelles and those of the chain segments. On the subsegmental length scale (high *q* in the NSE data; *q* > 0.08 Å^–1^, *t* < 100 ns), *S*(*q*, *t*)/*S*(*q*, 0) are almost identical within the experimental resolution for these three surfactants (see Figures 1 and S1). At this length scale, ca. 80 Å, the dynamics are mainly attributed to fluctuations of the micelle cross section, which is similar for the three systems from a structural point of view (see Table 1).^12,15^ Significant differences in *S*(*q*, *t*)/*S*(*q*, 0) are only observed at the lowest *q*-values in the NSE data (*q* < 0.0205 Å^–1^, *t* > 200 ns). From the analysis of the data, the relaxation rates (Γ(*q*)) were calculated using eq 1. The results are presented in Figure 2. Importantly, the relaxation time associated with micelle breakage is much longer than the probed time scale using NSE, and thus the micelles can be regarded to effectively behave as unbreakable chains.^2,12,15^

For the three surfactants, the cross-sectional dynamics can be described using the 2/β slope at high *q* (>0.08 Å^–1^), followed by a subtle increase in Γ(*q*) at intermediate *q* compared to the expected values from the 2/β slope and a drop in the relaxation rates at the lowest *q*-values (<0.03 Å^–1^) (see Figures 2 and S3). These deviations in the Γ(*q*) curves from the expected 2/β slope, as predicted for semiflexible chains,^19^ could be attributed to restricted dynamic processes associated to interchain interactions at this longer length scale. For the high *q* expansion of the NSE data, the relaxation rates of the system are the same within the limits of error. Only significant differences in Γ(*q*) can be observed at the lowest *q*-value, 0.0143 Å^–1^ (see Figure S2). At this length scale, ca. 400 Å, the relaxation rates follow the trend Γ(0.0143 Å^–1^)_β-C_16_G_2__ ≅ Γ(0.0143 Å^–1^)_α-C_16_G_2__ < Γ(0.0143 Å^–1^)_β-C_16_-1G_2__. This agrees with the SANS characterization as the β-C_16-1_G_2_ micelles were previously observed to be more flexible (i.e., shorter persistence length, see Table 1) than those of the saturated analogues, thus showing faster dynamics in the segmental domain.^12,15^ Also, extensional rheology revealed that β-C_16-1_G_2_ forms longer capillary columns than the saturated analogue β-C_16_G_2_ when a sample is gradually separated between two plates.^12^ This elongational flow has also been related to the presence of more flexible 1D macromolecules.^20,21^

From these results, the segmental diffusion coefficient, *D*_G_, is determined using
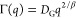
2with the β values obtained from the previous analysis of the data. The results are presented in Table 2. This approach to analyze the data assumes that the nanoscopic dynamics probed in NSE can be simplified using a single diffusion coefficient. Thus, the resulting *D*_G_ accounts for the contributions from the cross-sectional and segmental micelle dynamics. From *S*(*q*, *t*)/*S*(*q*, 0) and Γ(*q*) curves, we see that the results at a high *q* are very similar, confirming that the dynamics are similar at short length scales and Fourier times, attributed to cross-sectional fluctuations. The main differences are observed only at low *q*, where we identify changes in the *S*(*q*, *t*)/*S*(*q*, 0) curves, the β-values, and Γ(*q*) for different surfactants. Combining these results, we confirm that the differences in the relaxation dynamics between the micelles of the geometrically varied surfactants occur in the segmental length scale.

**Table 2:** Calculated Diffusion Coefficients for the Data Included in Figure 1

surfactant	*D*_G_ (Å^8/3^ ns^–1^)^a^	*D*_1_ (Å^2^ ns^–1^)^b^	*D*_2_ (×10^–4^ Å^2^ ns^–1^)^b^
α-C_16_G_2_	8.68 ± 0.04	0.99 ± 0.08	
β-C_16_G_2_	8.48 ± 0.09	2.01 ± 0.12	0.716 ± 0.060
β-C_16-1_G_2_	11.54 ± 0.18	2.59 ± 0.15	20.31 ± 1.07

a *D*_G_ corresponds to the nanoscopic diffusion as calculated from the NSE data using eq 2.

b *D*_1_ and *D*_2_ are the diffusion coefficients associated to the fast and slow relaxation modes of the network, respectively, calculated from the DLS data using eqs 3 and 4.

The *D*_G_ values show that the fastest diffusion probed using NSE is observed for β-C_16-1_G_2_ and the slowest for β-C_16_G_2_. It should be noted that fits are strongly influenced by Γ(*q*) at the lowest *q*-value, 0.0143 Å^–1^, and further differences in the segmental dynamics of these systems are possibly hidden in the inaccessible *q*-range of these experiments, i.e., 0.00238 Å^–1^ < *q* < 0.0143 Å^–1^. Considering the previous observations that the differences in the dynamics concentrate at the segmental length scale, the higher *D*_G_ value for β-C_16-1_G_2_ potentially relates to the more flexible chains. The β-C_16_G_2_ segments were found to be slightly less mobile than those of α-C_16_G_2_ despite having identical chemical composition. The differences in the micelle dynamics at the segmental length scale can be rationalized in terms of monomer packing, where a tighter monomer packing promoted by hydrogen bonding and hydrophobic interactions between neighboring monomers leads to the formation of less flexible micelles.^15,22^ In contrast, an increased micelle flexibility can be attributed to the less ordered and more dynamic packing of the unsaturated tails inside the micelle.^12^

The mesoscopic relaxations were characterized using a three-dimensional (3D) light scattering instrument from LS Instruments. For semidilute and concentrated solutions of flexible or semiflexible particles, two relaxation modes are commonly found in DLS measurements. The fast mode (1), usually referred to as the breathing mode, is related to the compressibility in the system. It originates from the compression and relaxation of the network and has a diffusive *q*^2^ dependence. The slow mode (2) is related to the motion of the chains due to topological constraints in the mesoscopic scale. This mode is strongly affected by the increased interaction due to crowding, causing slower diffusion with increasing concentration in semidilute and concentrated solutions.^3,23,24^ Thus, the dynamics of the slow mode can be related to the viscosity in the system. The relaxation mode can be probed from the intensity autocorrelation function, *g*_2_(*q*, *t*), using the following equation
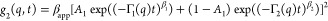
3where β_app_ is the coherent factor, *A*_1_ is the amplitude contribution of the fast mode, Γ_1_(*q*) and Γ_2_(*q*) are the fast and slow relaxation rates, and β_1_ and β_2_ are the stretched exponentials attributed to each mode.

For shorter micelles, where no entanglements are present, the large-scale dynamics can usually be described using one relaxation mode attributed to micellar diffusion^25^
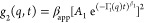
4The intensity autocorrelation functions and the best fits at different measured *q*-values for the three surfactants are presented in Figure 1d–f. The relaxation rates vs *q* are presented in Figure 2.

The mesoscopic dynamics show more prominent differences between the surfactants than those at the segmental length scale. While α-C_16_G_2_ shows a single relaxation mode, both β-C_16_G_2_ and β-C_16-1_G_2_ show two relaxations in the time frame investigated here. This distinct behavior is well correlated to the structural features of the micelles: α-C_16_G_2_ forms short cylindrical micelles that present translational diffusion; the β-anomers form an entangled network of WLM with the associated cooperative diffusion and self-diffusive motions.^3,12,15,26^ All of the relaxation rates show a *q*^2^ dependance, as expected.^23,27^

The stretched exponent for the fast relaxation mode, β_1_, is >0.9 for the three systems. This shows a narrow distribution of relaxation time for these processes. For α-C_16_G_2_, this indicates that the micellar distribution is relatively monodisperse and, thus, the dynamics follow a narrow distribution of translational diffusion coefficients. For the WLM-forming surfactants β-C_16_G_2_ and β-C_16-1_G_2_, the fast mode also shows a narrow distribution of diffusion coefficients associated to the breathing dynamics of the network. However, the slow mode shows a wide distribution of relaxation times associated with the self-diffusion of the network, with a stretched exponent around 0.4. The increased entanglement of the WLM, as well as the polydispersity of the contour length and persistence length of the micelles, could be responsible for the broad distribution of relaxation times and the low β_2_ values.^12,15^ Also, it should be noted that the amplitude of the fast mode increases with *q* (see Tables S3–S5), as expected for the breathing dynamics. From the fitted relaxation rates, the diffusion coefficients were calculated using the equation
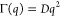
5The obtained results are presented in Table 2. In the length scale probed by the DLS experiment (>2000 Å) and as expected from the micellar shape, the α-C_16_G_2_ dynamics relate to the translational diffusion of the micelles.^15^ For the systems forming WLM, the cooperative diffusion is faster than the translational diffusion of α-C_16_G_2_. This increase of the cooperative diffusion in the entanglement regime is a known phenomenon and is related to the increase in compressibility. Since the entanglement points are getting closer with increasing concentration, the system responds faster to any compression. As such, the structural fluctuations are faster for more entangled networks and the measured cooperative diffusion is faster than the translational diffusion in the dilute regime.^28,29^ In the slow mode, the differences become more pronounced and the diffusion of β-C_16_G_2_ is slower than the unsaturated surfactant by at least one order of magnitude. Thus, the network dynamics (extracted from the DLS data, 5.48 × 10^–4^ Å^–1^ < *q* < 2.38 × 10^–2^ Å^–1^) correlate with the segmental diffusion (extracted from the NSE data, 0.014 Å^–1^ < *q* < 0.166 Å^–1^) for the two WLM-forming surfactants, where β-C_16-1_G_2_ is more dynamic than the saturated analogue at the explored length and time scales.

When attempting to relate the diffusion modes to the rheology of each system, previous investigations showed that higher values for the fast-mode diffusions and lower values for the slow-mode diffusions are associated with longer relaxation times.^3^ Here, it is observed that the faster diffusion of β-C_16-1_G_2_ WLM is associated with larger values of the characteristic rheological parameters, η_0_ and *G*′ ∩ *G*″, and longer relaxation times than the β-C_16_G_2_ WLM.^12,15^ From a structural point of view, the stiffer micelles are potentially expected to have fewer entanglement points. This reasoning can be extended to an infinitely stiff rod, which would show the minimum possible entanglements at any given length. On the other end, more flexible micelles of similar contour length offer more entanglement points, thus increasing the characteristic rheological parameters and the relaxation time.^30^

The intersection between the extrapolation of the high *q* relaxation rates in the NSE data (*q*^2/β^ scaling) to the fast-mode relaxation rates in the DLS data (*q*^2^ scaling) is expected to occur at *q* ≅ 2π*l*_p_^–1^, where *l*_p_ is the persistence length. Thus, this approach can be used to provide an approximate value of *l*_p_ from the topological dynamics of the system. The cross-over occurs at ca. 0.020 Å^–1^ for α-C_16_G_2_, ca. 0.023 Å^–1^ for β-C_16_G_2_, and ca. 0.037 Å^–1^ for β-C_16-1_G_2_. These *q*-values relate to an approximated value of *l*_p_ of 312 Å for α-C_16_G_2_, 270 Å for β-C_16_G_2_, and 173 Å for β-C_16-1_G_2_, as determined from the characterization of the dynamics of the system. This shows that *l*_p_ varies as β-C_16-1_G_2_ < β-C_16_G_2_ < α-C_16_G_2_, with α-C_16_G_2_ being the stiffest micelles and β-C_16-1_G_2_ the most flexible micelles of those investigated here. Thus, this agrees with the results from the structural investigations performed using SANS, as seen in Table 1.^11,12,15^ Note that the *l*_p_ value could not be directly determined using SANS for the α-C_16_G_2_ micelles, but the results for the β-C_16_G_2_/α-C_16_G_2_ mixtures showed that micelles were stiffer at high ratios of α-C_16_G_2_ than those of β-C_16_G_2_.^11,15^

## Conclusions

In summary, we have shown how the topological dynamics of micelles formed by compositionally identical (or similar) sugar-based surfactants are connected to their hierarchical structure. The similarity in the micellar cross section signifies that the dynamics are similar at short length scales for the three surfactants studied here. However, these begin to differentiate at the segmental length scale. This is potentially attributed to the flexibility of the micelle, where the inefficient packing caused by kinks in the structure of the β-C_16-1_G_2_ monomer leads to faster segmental diffusion compared to that of the tightly packed β-C_16_G_2_. At the mesoscopic scale, the differences become more pronounced. For shorter cylindrical micelles of α-C_16_G_2_, only one relaxation mode is observed, and this is attributed to the translational diffusion of those micelles. In the case of WLM, the higher conformational flexibility of β-C_16-1_G_2_ prompts a faster network breathing and, importantly, much faster and defined slow mode than the β-C_16_G_2_ micelles.

The differences in micelle dynamics also extend to the macroscopic scale, where the nonentangled, diffusing α-C_16_G_2_ micelles relate to low viscosity, Newtonian fluids, whereas the entangled networks of WLM result in shear-thinning fluids. Interestingly, β-C_16-1_G_2_ leads to more viscous systems, although more dynamic in the mesoscopic scale. This could be attributed to the formation of a more entangled network that follows different relaxation pathways relative to its saturated counterpart. Therefore, it is concluded that the packing of the monomers, controlled by the molecular architecture of the compositionally identical surfactants, dictates the structure and dynamics of the micelles on the nano length scale and the nanosecond time frame that, as we demonstrate, control the rheological properties of the system.

## Supporting Information Available

Materials and methods; analysis of the NSE stretched exponent; and results from the analysis of the DLS data (PDF)
