# Supplementary material for: Topological Dynamics of Micelles Formed by Geometrically Varied Surfactants
Source: Langmuir. 2022 Aug 1;38(33):10075–80. doi: 10.1021/acs.langmuir.2c00230 (PMC9404537; doi:10.1021/acs.langmuir.2c00230)
Supplement: Supplementary file 1 — la2c00230_si_001.pdf [file la2c00230_si_001.pdf]

Supporting information for:
Topological dynamics of micelles formed by geometrically varied surfactants

Adrian Sanchez-Fernandez,[a]* Johan Larsson,[b] Anna Leung,[c] Peter Holmqvist,[d] Orsolya Czakkel,[e] Tommy Nylander,[d] Stefan Ulvenlund,[f] Marie Wahlgren.[a]
[a] Food Technology, Engineering and Nutrition, Lund University, Box 124, 221 00 Lund, Sweden.
[b]Biofilms Research Center for Biointerfaces and Department of Biomedical Science, Faculty of Health and Society, Malmö University, Per Albin Hanssons Väg 35, 21432 Malmö, Sweden.
[c]European Spallation Source ERIC, P.O. Box 176, 221 00 Lund, Sweden.
[d]Physical Chemistry, Department of Chemistry, Lund University, Box 124, 221 00 Lund, Sweden.
[e]Institut Laue-Langevin, 71 Avenue des Martyrs, 38000 Grenoble, France.
[f]EnzaBiotech AB, Scheelevägen 22, 22363 Lund, Sweden.
*adriansanchez.fernandez@usc.es

Experimental

n-Hexadecyl-β-D-maltoside (Anatrace Inc., >97%, β-$C_{16}G_2$) and n-Hexadecyl-α-D-maltoside (Ramidus AB, >97%, α-$C_{16}G_2$) were used as received. Palmitoleyl-b-D-maltoside ((Z)-Hexadec-9-en-1-yl-β-D-maltoside, β-$C_{16\text{-}1}G_2$) was synthesized via a Koenigs-Knorr glycosylation reaction as previously reported.[1] D_2O (99.9% D) was supplied by Sigma-Aldrich. Samples were prepared at a surfactant concentration of 100 mM by dissolving the required amounts of surfactant in D_2O and subsequent equilibration at 50 ˚C under stirring for 8 hours.

Neutron spin echo (NSE) experiments were performed on IN15 at the Institut Laue-Langevin, France. The instrument was set up to cover the momentum transfer, q, range of 0.014 $Å^{-1} \leq q \leq$ 0.166 $Å^{-1}$. The q-values probed in the experiment are listed in Table S1. By using 5 different incident wavelength of the neutron beam (17 Å, 13.5 Å, 12 Å, 8 Å, and 6 Å), the Fourier-time range of 4-952 ns has been covered. The resolution functions of the instruments were determined for each experimental setup using the elastic scattering of graphite. The resulting intermediate scattering functions were corrected for the D_2O background dynamics. Samples were loaded in a temperature-controlled sample changer set at 50 ˚C for the measurements.

Table S1 q values in $Å^{-1}$ covered in the NSE experiment.

0.0143	0.0377	0.0973
0.0205	0.0453	0.1109
0.0218	0.0562	0.1296
0.0268	0.0670	0.1478
0.0298	0.0836	0.166

The intermediate scattering functions at short Fourier times and a comparison of the intermediate scattering functions at various q values for the three surfactants are presented in Fig. S1.

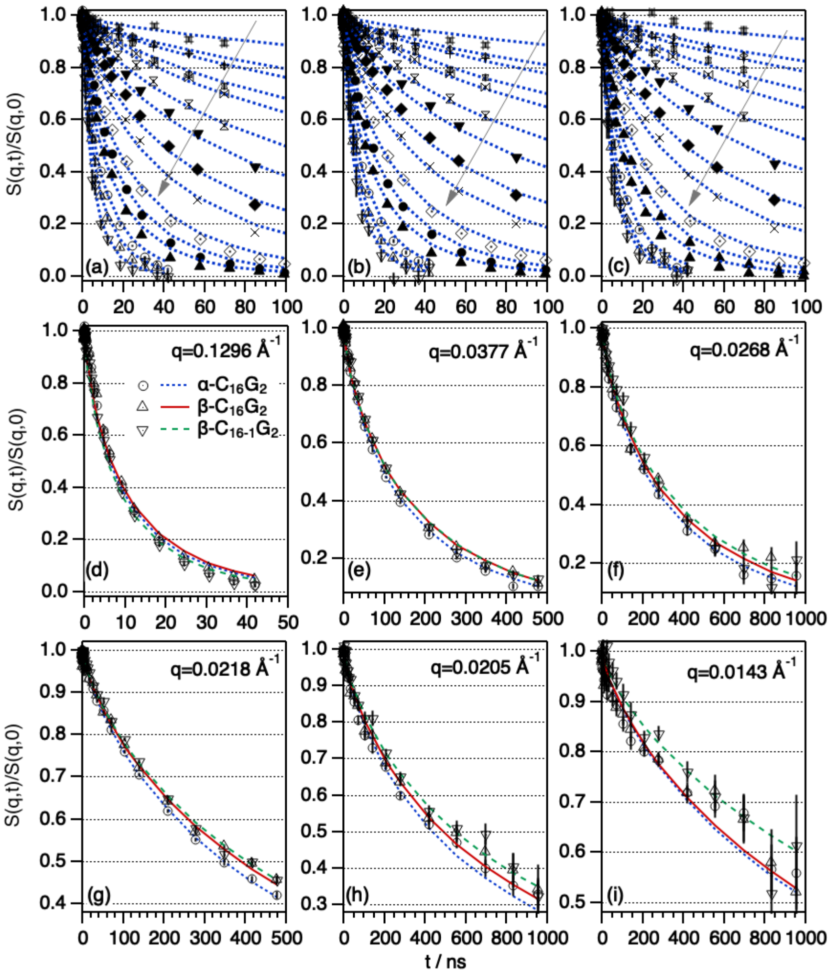

Fig. S1 Normalized intermediate scattering functions and best fits for 100 mM of (a) α-$C_{16}G_2$, (b) β-$C_{16}G_2$, and (c) β-$C_{16\text{-}1}G_2$ at 50 °C in D_2O, covering a q-range between 0.014 $Å^{-1}$ and 0.166 $Å^{-1}$. The q-values increase in the direction of the arrows. (d-i) Comparison of

the normalized intermediate scattering functions and models for the three surfactants at different q values, as indicated in each panel. Experimental data are presented as markers and models are presented as lines, as indicated in the legend of panel (d). Where not visible, error bars are within the markers.

Dynamic light scattering measurements (DLS) were performed on a 3D-DLS Spectrometer (LS Instruments Gmbh, Switzerland) equipped with 660 nm Cobolt laser with a maximum power of 100 mW covering a q-range of 5.48×10^{-4} Å^{-1} $\leq q \leq 2.38\times10^{-2}$ Å^{-1}. The q-values probed in the experiment are listed in Table S2. The scattered light was detected within an angular range of 25 and 140˚ by two avalanche photodiodes. The intensity was processed by LS instrument correlators and the resulting intensity auto-correlation function, $g^2(q,t) - 1$ was exported for analysis. The samples were prepared in a 10 mm sample tube and heated to 50 ˚C. Due to the high viscosity of the samples air bubbles were trapped in the sample and were removed by first gently centrifuging the sample, after which it was kept at 50 ˚C for 1h prior measurement. Subsequently, they were loaded into a temperature-controlled index matching vat at 50 ˚C and measured.

Table S2 q values in Å^{-1} covered in the DLS experiment.

0.00054809	0.00116929	0.00171081	0.00213573
0.00065541	0.00126616	0.00179062	0.00219305
0.00076148	0.00136061	0.00186702	0.00224619
0.0008661	0.00145248	0.00193987	0.00229506
0.00096908	0.00154158	0.00200902	0.00233955
0.00107020	0.00162774	0.00207435	0.00237960

Validation of the segmental relaxation mode

In the model proposed by Zilman and Granek, the normalised intermediate scattering function (NISF) can be described using a single stretched exponential function, where the stretch exponent (β) relates to the relaxation modes of the system. As such, β=3/4 describes the relaxation from one-dimensional particles, e.g., worm-like micelles (WLM), and β=2/3 matches the relaxation dynamics of two-dimensional objects, e.g., membranes.[2] As branched structures provide additional relaxation mechanisms to WLM solutions, the segmental dynamics are similar to those from 2D objects.[3] To test the relaxation mechanism of the systems investigated here, we have attempted to fit the data using both stretched exponents. Data and fits are presented in Fig. S2, together with the quality of each fit represented by the χ^2 parameter.

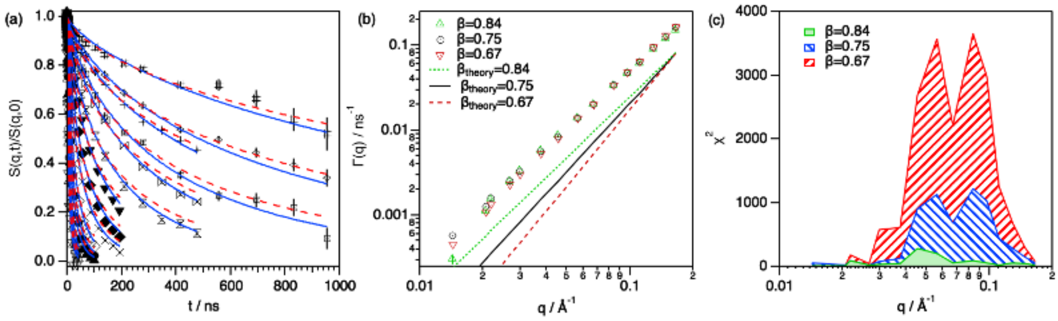

Fig. S2 (a) Experimental normalised intermediate scattering function for 100 mM β-$C_{16-1}G_2$ at 50 ˚C and fits using different value of β: (blue solid line) 2/3, and (red dashed line) 3/4. (b) Fitted relaxation rates and (c) χ^2 values as a function of q for each stretched exponent, as indicated in the legend of b.

The validation of the different stretched exponents showed that the fits obtained using β=3/4 are significantly better than those obtained using β=2/3, as the χ^2 values are consistently lower in the entire q-range for this system. This indicates that the relaxation dynamics of the sugar-based surfactant micelles are best described using the mechanism for one-dimensional supramolecular assemblies. It is also observed that by using the fitted stretched exponents, the quality of the fit significantly improves. Therefore, the fitted β was selected for the subsequent analysis of the data reported in this manuscript.

The analysis of the NISF using the fitted β still leads to certain disagreement between the trend described by the data and the expected slope for β=0.84 (See Fig. S3). This disagreement becomes more prominent in the low q regime of the NSE data and is discussed in the main text.

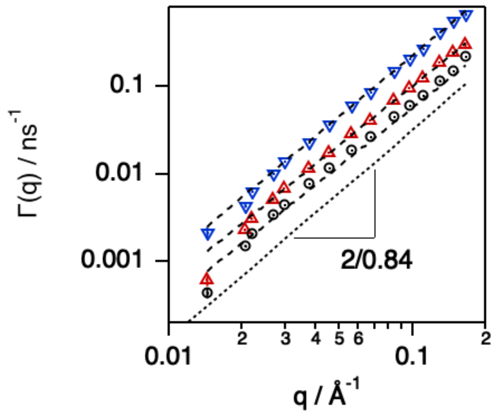

Fig. S3 Calculated Γ(q) vs. q for 100 mM α-$C_{16}G_2$, β-$C_{16}G_2$, and β-$C_{16-1}G_2$ at 50 ˚C (as indicated in the graph legend) fitted β=0.84. Data and models are offset for clarity: ×2 for β-$C_{16}G_2$ and ×4 for β-$C_{16-1}G_2$.

DLS results

Table S3 Results from the analysis of the DLS data for 100 mM α-$C_{16}G_2$ at 50 ˚C using the single stretched exponential function (Eq. 4).

q / Å^{-1}	A_1	$β_1$	Γ(q) / ×10^{-7} ns^{-1}
0.00054809	0.965±0.007	0.837±0.004	2.93±0.03
0.00065541	0.968±0.006	0.844±0.004	4.33±0.04
0.00076148	0.967±0.002	0.846±0.003	5.90±0.04
0.0008661	0.967±0.024	0.847±0.004	7.61±0.07
0.00096908	0.966±0.031	0.855±0.003	9.75±0.08
0.0010702	0.967±0.073	0.854±0.003	11.87±0.09
0.00116929	0.970±0.021	0.847±0.004	13.71±0.14
0.00126616	0.969±0.030	0.852±0.003	16.36±0.13
0.00136061	0.970±0.062	0.848±0.003	18.86±0.15
0.00145248	0.969±0.08	0.849±0.003	21.57±0.17
0.00154158	0.969±0.078	0.847±0.003	24.10±0.19
0.00162774	0.969±0.010	0.840±0.004	26.59±0.22
0.00171081	0.968±0.008	0.841±0.003	29.25±0.23
0.00179062	0.968±0.078	0.834±0.003	31.49±0.26
0.00186702	0.967±0.009	0.834±0.003	34.32±0.27
0.00193987	0.967±0.008	0.832±0.003	37.19±0.29
0.00200902	0.968±0.013	0.830±0.003	39.60±0.33
0.00207435	0.968±0.003	0.827±0.003	41.90±0.33
0.00213573	0.968±0.006	0.820±0.003	43.86±0.36
0.00219305	0.972±0.007	0.807±0.005	44.38±0.61
0.00224619	0.968±0.007	0.815±0.004	47.81±0.43
0.00229506	0.968±0.013	0.811±0.003	49.57±0.43
0.00233955	0.968±0.009	0.806±0.004	51.06±0.48
0.0023796	0.969±0.008	0.802±0.004	52.47±0.48

Table S4 Results from the analysis of the DLS data for 100 mM β-$C_{16}G_2$ at 50 ˚C using the single stretched exponential function (Eq. 3).

q / $Å^{-1}$	A_1	$β_1$	$Γ_1(q)$ / $×10^{-7}$ ns^{-1}	$β_2$	$Γ_2(q)$ / $×10^{-10}$ ns^{-1}
0.00054809	0.479±0.016	0.982±0.026	6.45±0.11	0.251±0.02	4.00±79.34
0.00065541	0.381±0.009	0.980±0.019	9.29±0.16	0.31±0.017	1.86±1.68
0.00076148	0.349±0.004	0.979±0.010	12.36±0.13	0.341±0.008	0.71±0.14
0.0008661	0.450±0.003	0.889±0.014	14.31±0.25	0.63±0.019	3.45±0.34
0.00096908	0.516±0.006	0.976±0.007	20.31±0.13	0.283±0.01	7.82±15.13
0.0010702	0.327±0.004	0.991±0.010	23.38±0.20	0.254±0.006	0.42±0.24
0.00116929	0.444±0.002	0.871±0.010	26.57±0.34	0.517±0.012	2.48±0.24
0.00126616	0.431±0.003	0.893±0.009	32.48±0.34	0.374±0.008	1.69±0.37
0.00136061	0.535±0.002	0.900±0.006	38.54±0.27	0.374±0.008	5.56±1.69
0.00145248	0.562±0.003	0.857±0.009	42.82±0.53	0.445±0.014	4.69±1.40
0.00154158	0.606±0.005	0.873±0.008	47.56±0.57	0.398±0.018	12.14±8.00
0.00162774	0.575±0.004	0.844±0.012	51.50±0.95	0.484±0.022	7.14±2.24
0.00171081	0.585±0.005	0.878±0.007	58.02±0.53	0.295±0.012	9.18±16.17
0.00179062	0.470±0.003	0.817±0.010	57.65±0.87	0.376±0.01	1.00±0.28
0.00186702	0.517±0.003	0.823±0.009	65.43±0.88	0.343±0.011	1.09±0.55
0.00193987	0.561±0.003	0.830±0.008	73.49±0.96	0.365±0.011	2.23±1.15
0.00200902	0.635±0.006	0.844±0.009	78.86±1.15	0.349±0.019	12.44±19.04
0.00207435	0.602±0.006	0.852±0.008	81.45±0.92	0.257±0.013	6.61±55.26
0.00213573	0.580±0.004	0.835±0.005	87.68±1.01	0.291±0.01	4.50±8.45
0.00219305	0.641±0.006	0.818±0.009	90.66±1.41	0.325±0.017	6.42±16.12
0.00224619	0.608±0.005	0.819±0.009	94.51±1.41	0.297±0.014	4.87±9.43
0.00229506	0.647±0.006	0.813±0.009	98.59±1.66	0.35±0.018	6.70±13.28
0.00233955	0.608±0.006	0.810±0.010	102.14±1.72	0.274±0.015	3.17±18.14
0.0023796	0.624±0.007	0.813±0.006	106.05±1.63	0.234±0.151	2.13±54.59

Table S5 Results from the analysis of the DLS data for 100 mM β-$C_{16\text{-}1}G_2$ at 50 ˚C using the single stretched exponential function (Eq. 3).

q / $Å^{-1}$	A_1	$β_1$	$Γ_1(q)$ / $×10^{-7}$ ns^{-1}	$β_2$	$Γ_2(q)$ / $×10^{-10}$ ns^{-1}
0.00054809	0.227±0.007	0.981±0.022	7.95±4.17	0.380±0.009	16.07±8.11
0.00065541	0.387±0.005	0.947±0.006	11.13±3.77	0.504±0.011	22.75±2.11
0.00076148	0.390±0.007	0.929±0.010	15.81±5.12	0.468±0.010	11.60±2.06
0.0008661	0.379±0.007	0.940±0.012	20.07±7.34	0.459±0.011	12.77±2.74
0.00096908	0.382±0.004	0.928±0.007	25.13±7.31	0.485±0.008	13.41±1.42
0.0010702	0.445±0.007	0.929±0.009	32.18±10.40	0.447±0.011	16.13±4.89
0.00116929	0.507±0.003	0.919±0.004	37.35±8.27	0.487±0.009	37.48±5.64
0.00126616	0.581±0.004	0.917±0.005	43.94±12.42	0.512±0.016	79.61±19.28
0.00136061	0.564±0.004	0.922±0.004	50.35±12.18	0.497±0.012	60.62±13.00
0.00145248	0.577±0.004	0.917±0.004	57.32±15.56	0.530±0.014	82.72±16.75
0.00154158	0.590±0.004	0.914±0.004	63.20±18.59	0.487±0.017	78.10±19.31
0.00162774	0.591±0.005	0.918±0.005	70.27±19.40	0.469±0.016	95.62±33.49
0.00171081	0.600±0.004	0.915±0.004	77.05±20.56	0.460±0.016	97.22±31.31
0.00179062	0.631±0.005	0.912±0.005	83.28±22.82	0.475±0.020	156.98±68.21
0.00186702	0.614±0.004	0.906±0.004	89.97±24.68	0.487±0.017	109.42±33.84
0.00193987	0.597±0.006	0.903±0.005	94.03±27.17	0.434±0.016	90.24±49.83
0.00200902	0.605±0.006	0.902±0.005	100.94±28.38	0.432±0.016	110.44±64.15
0.00207435	0.607±0.005	0.898±0.005	106.68±30.67	0.428±0.016	99.46±56.96
0.00213573	0.621±0.005	0.890±0.005	113.19±33.67	0.436±0.018	102.47±61.61
0.00219305	0.629±0.006	0.889±0.006	118.38±38.32	0.430±0.021	120.66±87.67
0.00224619	0.627±0.008	0.892±0.006	122.78±37.31	0.416±0.020	158.31±168.35
0.00229506	0.624±0.007	0.892±0.006	128.45±41.56	0.412±0.021	155.26±149.14
0.00233955	0.636±0.009	0.882±0.007	131.67±47.65	0.427±0.024	169.46±219.48
0.0023796	0.606±0.009	0.892±0.007	136.76±46.6	0.365±0.020	126.78±185.78

References

1. Larsson, J.; Leung, A. E.; Lang, C.; Wu, B.; Wahlgren, M.; Nylander, T.; Ulvenlund, S.; Sanchez-Fernandez, A. Tail unsaturation tailors the thermodynamics and rheology of a self-assembled sugar-based surfactant. *J. Colloid Interface Sci.* **2021,** *585*, 178-183.
2. Zilman, A. G.; Granek, R. Undulations and Dynamic Structure Factor of Membranes. *Phys. Rev. Lett.* **1996,** *77* (23), 4788-4791.
3. Calabrese, M. A.; Wagner, N. J. Detecting Branching in Wormlike Micelles via Dynamic Scattering Methods. *ACS Macro Letters* **2018,** *7* (6), 614-618.
